# Construction, Identification and Analysis of the Interaction Network of African Swine Fever Virus MGF360-9L with Host Proteins

**DOI:** 10.3390/v13091804

**Published:** 2021-09-10

**Authors:** Bo Yang, Dajun Zhang, Xijuan Shi, Chaochao Shen, Yu Hao, Ting Zhang, Jinke Yang, Xingguo Yuan, Xuehui Chen, Dengshuai Zhao, Huimei Cui, Dan Li, Zixiang Zhu, Hong Tian, Fan Yang, Haixue Zheng, Keshan Zhang, Xiangtao Liu

**Affiliations:** State Key Laboratory of Veterinary Etiological Biology, National Foot-and-Mouth Disease Reference Laboratory, Lanzhou Veterinary Research Institute, Chinese Academy of Agricultural Science, Lanzhou 730046, China; 2019107002@njau.edu.cn (B.Y.); vetzdj129@163.com (D.Z.); shixijuan103@163.com (X.S.); shenchaochao1989@163.com (C.S.); zghaoyu0616@163.com (Y.H.); 18793598414@163.com (T.Z.); y18860362893@163.com (J.Y.); y15390692865@163.com (X.Y.); cxh18894354570@163.com (X.C.); zds523072053@163.com (D.Z.); chm18809481412@163.com (H.C.); lidan@caas.cn (D.L.); zhuzixiang@caas.cn (Z.Z.); tianhong@cass.cn (H.T.); yangfan02@cass.cn (F.Y.); liuxiangtao@caas.cn (X.L.)

**Keywords:** African swine fever virus, MGF360-9L, protein interactions network, PSMA3, PSMC1

## Abstract

African swine fever virus (ASFV) is prevalent in many countries and is a contagious and lethal virus that infects pigs, posing a threat to the global pig industry and public health. The interaction between the virus and the host is key to unlocking the mystery behind viral pathogenesis. A comprehensive understanding of the viral and host protein interaction may provide clues for developing new antiviral strategies. Here, we show a network of ASFV MGF360-9L protein interactions in porcine kidney (PK-15) cells. Overall, 268 proteins that interact with MGF360-9L are identified using immunoprecipitation and liquid chromatography–mass spectrometry (LC-MS). Accordingly, gene ontology (GO) and Kyoto Encyclopedia of Genes and Genomes (KEGG) enrichment analyses were conducted, and the protein–protein interaction (PPI) network was created. It was speculated that the cellular proteins interacting with MGF360-9L are involved in protein binding, metabolism, and the innate immune response. Proteasome subunit alpha type (PSMA3), 26S protease regulatory subunit 4 (PSMC1), autophagy and beclin 1 regulator 1 (AMBRA1), and DEAD-box helicase 20 (DDX20) could interact with MGF360-9L in vitro. PSMA3 and PSMC1 overexpression significantly promoted ASFV replication, and MGF360-9L maintained the transcriptional level of PSMA3 and PSMC1. Here, we show the interaction between ASFV MGF360-9L and cellular proteins and elucidate the virus–host interaction network, which effectively provides useful protein-related information that can enable further study of the potential mechanism and pathogenesis of ASFV infection.

## 1. Introduction

African swine fever (ASF) is a highly pathogenic infectious disease caused by the African swine fever virus (ASFV), the only member of the *Afarviridae* family. It causes high morbidity and mortality in domestic pigs and wild boars [1,2]. ASF cases first occurred in Africa and then spread to Europe, entered China in 2018, and swept through the country within one year [3,4]. As there are no effective and specific drugs or commercial vaccines at present, stringent prevention and control measures to conduct early laboratory diagnosis and culling of animals infected with ASFV and those with suspected infections are generally and internationally accepted. This has caused substantial economic losses in countries where outbreaks have occurred and posed a significant threat to global food safety and supply [5,6]. ASFV is a large enveloped virus with a large (between 180 and 190 kb) double-stranded DNA genome that encodes more than 150 open reading frames (ORFs) [7,8]. ASFV is the only DNA arbovirus, and has a symmetrical icosahedral particle with a diameter of approximately 200 nm [9]. Despite researchers’ efforts, due to the large number of proteins encoded by ASFV, there are still many proteins whose functions are unknown.

Many proteins encoded by ASFV can evade an immune defense through various strategies. Host cell protein expression systems and cytokine transcription can be regulated by DP71L, A238L, and pE66L [10,11]. Programmed cell death is regulated by ASFV E183L, A179L, A224L, and EP153R [12,13,14], whereas MGF360, MGF505/530, and I329L inhibit the type I IFN signal pathway [15,16]. ASFV MGF360 is located in both left and right variable ends of the genome. It has been reported that MGF360 plays an essential role in the host range of macrophages and ASFV pathogenesis in pigs [17]. The MGF360 A276R gene can inhibit IFN-β expression by targeting IRF3 [18], and MGF360-12L inhibits type I IFN production by blocking the interaction between the importinα and NF-κB signaling pathway [19]. Understanding the functions of even more ASFV genes enables the production of an experimental attenuated ASFV candidate vaccine. Finding and understanding the genes that play a crucial role in ASFV virulence is important for developing and using gene-operated vaccines. A part of the ASFV gene was successfully deleted from the ASFV genome, and it was confirmed that the deleted recombinant viruses, such as I177L, DP148R, 9GL, and UK, were attenuated in pigs [20,21,22,23]. Also, deleting or interrupting some MGF360 and MGF530/505 genes, including MGF360-9L, from an ASFV virulent isolate reduces virulence in domestic pigs and induces a protective response [24,25]. MGF360-9L, an MGF360 member, exists widely in naturally isolated virulent strains of ASFV and is highly conserved in the same genotype, but there are significant differences at the 5′ end of the MGF360-9L gene among different ASFV genotypes. Previously, the prediction for the secondary structure of MGF360-9L showed that the α-helix accounted for 34.29%, the extended chain accounted for 30.57%, and the irregular crimp accounted for 35.14% [26].

During the infection process, the host’s innate immune and transcriptional and translation systems are reshaped by the virus–host interaction, which affects the virus’s ability to use the host to promote its reproduction [27]. ASFV replicates mainly in monocytes and macrophages; ASFV regulation of macrophage function is essential to its immune escape and pathogenesis [28]. The traditional screening method of host protein interaction is not enough to study the ASFV replication mechanism and pathogenesis in detail. Therefore, a combination of co-immunoprecipitation and LC-MS, a high-throughput screening method, is used to study the virus and host protein interaction and has been used to study various viruses, including H5N1 influenza A virus [29] and porcine circovirus type 2 [30]. However, it has neither been used in ASFV nor in a comprehensive analysis of MGF360-9L.

Therefore, to further understand the interaction between MGF360-9L and host proteins and understand the pathogenesis and immune escape mechanism of ASFV, we identified 268 proteins that might interact with MGF360-9L from PK-15 cells transfected with MGF360-9L plasmid using co-immunoprecipitation and mass spectrometry. Subsequently, bioinformatics analysis of these proteins was conducted, and a protein–protein interaction (PPI) network was constructed. It was identified that MGF360-9L interacts with four metabolism-related factors, PSMA3, PSMC1, AMBRA1, and DDX20 in vitro and that MGF360-9L may be involved in host metabolism regulation. It was determined that PSMA3 and PSMC1 promote ASFV replication. This study shows the basic interaction of MGF360-9L proteins in PK-15 cells. It provides a basis for further studies on the specific mechanism of MGF360-9L that is involved in the pathogenesis and immune escape of ASFV.

## 2. Materials and Methods

### 2.1. Cells and Virus

Porcine alveolar macrophage (PAM) was prepared using bronchoalveolar lavage as described above [31] and cultured in Roswell Park Memorial Institute medium containing 10% porcine serum. Cells were grown at 37 °C in a 5% CO_2_ atmosphere saturated with water vapor. PK-15, MA-104, and HEK-293T cells were cultured at 37 °C and 5% CO_2_ in Dulbecco’s modified Eagle medium with 10% fetal bovine serum (Gibco, Carlsbad, CA, USA), 100 μg/mL streptomycin, and 100 U/mL penicillin. MA-104 (microbiological associates-104) was a commercial cell line (Procell, Wuhan, China). The preservation institution of the MA-104 is: ECACC; 85102918.

ASFV isolate CN/GS/2018 propagates on PAM. Virus stocks were stored at −80 °C. Titration of the virus was performed by a hemadsorption assay; the results are presented as HAD_50_ per milliliter. ASFV and ASFV-WT used are ASFV isolate CN/GS/2018. ASFV-d360-9L is a strain produced by deleting the MGF360-9L gene based on the ASFV CN/GS/2018 strain. The ASFV-d360-9L detailed construction process can be found in the reference patent [32]. Lanzhou Veterinary Research Institute provided all ASFV strains.

### 2.2. Antibodies and Reagents

Mouse monoclonal antibody against Myc (2276S), β-Actin (3700S), and rabbit monoclonal antibody against Myc (13987S) and FLAG (14793S) were purchased from Shanghai Youningwei Biotechnology Co., Ltd. (Shanghai, China).

Mouse monoclonal antibody against GFP (66002-1-Ig), rabbit monoclonal antibody against GFP (50430-2-AP), HRP-Goat anti-Rabbit IgG (H + L) (SA00001-2), HRP-Goat anti-Mouse IgG (H + L) (SA00001-1), and protein G sepharose (17061801) were purchased from Lanzhou Lihe Biotechnology Co., Ltd. (Lanzhou, China). HRP-Goat anti-Mouse IgG (SE131) and HRP-Goat anti-Rabbit IgG (SE134) were purchased from Beijing Solarbio Science and Technology Co., Ltd. (Beijing, China).

### 2.3. Plasmid Construction and Cell Transfection

The ASFV isolate CN/GS/2018 MGF360-9L sequence was synthesized following gene synthesis technique and cloned into vectors pCDNA3.1 (+)-3*FLAG-C and pCDNA3.1(+)-EGFPC1. The Myc-PSMA3, Myc-PSMC1, Myc-AMBRA1, and Myc-DDX20 plasmid was constructed by inserting the ORF of PSMA3 (Gene ID: 100154408), PSMC1 (Gene ID: 100155274), and AMBRA1 (Gene ID: 100519700), and DDX20 (Gene ID: 100152786) into vector pCDNA3.1(+)-Myc-C (Wuhan GeneCreate Biological Engineering Co., Ltd., Wuhan, China).

To transfect the related plasmid, HEK-273T cells or MA-104 cells were inoculated onto the designated plate at the appropriate density according to the experimental scheme and grew to 80% confluence. The constructed plasmids were transfected into the cells with lipofectamine2000 transfection reagent (Invitrogen, Carlsbad, CA, USA). Lipofectamine reagent was diluted in Opti-MEM medium; in addition, DNA was diluted in Opti-MEM medium. After 5 min, the diluted DNA was added to the diluted Lipofectamine 2000 reagent. After incubation for 20 min, DNA–lipid complex was added to the cells.

### 2.4. LC-MS/MS

The experiments were performed on a Q Exactive mass spectrometer coupled with Easy nLC (Thermo Fisher Scientific, Waltham, MA, USA). The peptide mixture was loaded onto the C18 reversed-phase column (15 cm long and 75 μm inner diameter) packed in-house with RP-C18 5 μm resin in buffer A (0.1% formic acid in HPLC-grade water) and separated with a linear gradient of buffer B (0.1% formic acid in 84% acetonitrile) at a flow rate of 250 nL/min controlled by intelliflow technology over 60 min. MS data were acquired using a data-dependent top 10 method, dynamically choosing the most abundant precursor ions from the survey scan (300–1800 *m*/*z*) for HCD fragmentation. To determine if the target value is based on predictive automatic gain control (pAGC), dynamic exclusion duration was set to 20 s. Survey scans were acquired at a resolution of 70,000 at *m*/*z* 200, and the resolution for HCD spectra was set to 17,500 at *m*/z 200. The normalized collision energy was 27 eV, and the underfill ratio, which specifies the minimum percentage that the target value is likely to reach at maximum fill time, was defined as 0.1%. The instrument was run with its peptide recognition mode enabled.

### 2.5. LC-MS/MS Data Analysis

MS/MS spectra were searched using the MASCOT engine (Matrix Science, London, UK; v.2.2) against the UniProt Galagidae protein database (including 20,638 sequences, downloaded on v20200511). For protein identification, the following options were used: peptide mass tolerance = 20 ppm, MS/MS tolerance = 0.1 Da, enzyme = trypsin, missed cleavage = 2, fixed modification = carbamidomethyl (C), variable modification = oxidation (M), ion score > 20, and FDR < 0.01 at peptide and protein levels.

### 2.6. Construction and Analysis of PPI Network

Based on all datasets, the MGF360-9L-host protein interaction network was generated using Cytoscape v.3.7.1. To analyze the interaction between host proteins, a string database was used. Topological parameters and central measures of the network were calculated using a network analyzer tool in Cytoscape v.3.7.1. Pig protein–protein interaction analysis was also performed using the STRING database.

### 2.7. Protein Functional Enrichment Analysis

Gene ontology (GO) enrichment analysis was conducted using Cytoscape v.3.7.1, with a *p* value of <0.05. The Kyoto Encyclopedia of Genes and Genomes (KEGG) database was accessed using the KOBAS software via hypergeometric test, with a corrected *p*-value of <0.05 [18]. Enriched protein domain analysis was performed using FunRich (http://funrich.org/index.html, accessed on 2 December 2020).

### 2.8. Co-Immunoprecipitation

The related plasmids were transfected into HEK-293T cells using liposome 2000. Then, 24 h after transfection, the cells were lysed using a NP40 buffer containing PMSF for 1 h at 4 °C, and then subhected to ultrasound for 1.5 min with an interval of 5 s. The power rating of the sonicator was 125 W, the frequency was 20 kHz, and the output intensity was set to 30%. The cell lysate was then incubated overnight with the indicated antibody or control IgG at 4 °C. Consequently, samples were mixed with protein A/G agarose beads (Roche, Basel, Switzerland) for 3 h, washed with the NP40 buffer thrice, and boiled in a sodium dodecyl sulfate loading buffer.

### 2.9. SDS-PAGE and Immunoblotting Analyses

For western blotting, to isolate proteins using standard SDS-PAGE (80 V; 30 min, 120 V; 60 min). The protein was transferred to a nitrocellulose membrane (Pall, New York, NY, USA) (100 V 90 min), then sealed with 5% skim milk, washed with TBS containing 0.1% Tween 20 (TBST), incubated overnight with an antibody at 4 °C, washed three times with TBS containing 0.1% Tween 20, and incubated with the designated secondary antibodies. Finally, an electrochemiluminescence solution was added to the incubator, and the image was obtained using an Odyssey infrared imaging system.

### 2.10. Indirect Immunofluorescence Assay (IFA)

The corresponding expression plasmid was transfected into cells with lipofectamine 2000 or jetPRIME transfection reagent. At 24 h after transfection, the cells were fixed with 4% paraformaldehyde for 30 min, permeabilized with 0.2% TritonX-100 for 10 min, and blocked in 5% BSA for 1 h. Next, the cells were incubated with corresponding mAb for 8 h. Then, this was incubated with Alexa Fluor 488 anti-rabbit/mouse and Alexa Fluor 594 anti-rabbit/mouse lgG H&L (Abcam) for 2 h, and after stained with 4-methyl-6-phenylindole for 10 min. The samples were detected by the Leica SP2 confocal system (Leica Microsystems, Wetzlar, Germany) or the EVOS M5000 cell imaging system (Invitrogen, Carlsbad, CA, USA).

### 2.11. Real-Time qPCR

PAM was inoculated in a 6-well plate for 24 h and then infected with ASFV-WT (MOI = 0.1) or ASFV-d360-9L (MOI = 0.1) at 12 and 24 hpi, respectively. Samples were collected to extract total RNA. Total RNA was extracted from PAM using TRIzol reagent and reverse transcribed with PrimeScript RT kit (Takara, Otsu, Japan). qPCR was performed using the Power Up SYBR Green Master Mix on the ABI StepOnePlus system. All data were analyzed using the StepOnePlus software, and the relative mRNA level of these genes was normalized to porcine GAPDH mRNA level. In addition, the relative expression of mRNA was calculated based on the comparative cycle threshold (2^−ΔΔCT^) method [33]. The primer sequence information is presented in Table 1.

Samples were collected at a specified time after PAM was inoculated with ASFV. Real-time quantitative PCR was performed using the ASFV P72 gene as a target to detect the copy number of the ASFV genomic DNA. QIAamp DNA Mini Kits (Qiagen, Germany) were used to extract sample DNA, and then qPCR was performed using a Bio-Rad system. ASFV-P72-R: 5′-CTGCTCATGGTATCAATCTTATCGA-3′; ASFV-P72-F: 5′- GATACCACAAGATCAGCCGT-3′; Taqman: 5′-CCACGGGAGGAATACCAACCCAGTG-3′. The amplification conditions used were a preheating at 95 °C for 30 s, and 40 cycles of 95 °C for 5 s and 58 °C for 30 s. The quantity of the ASFV genome was calculated using the standard curve and was expressed as genome copies per milliliter.

### 2.12. Viral Titration (50% Hemadsorption Doses)

The anticoagulated whole blood of healthy pigs was washed with sterilized PBS (pH 7.2) containing 1% gentamicin. Next, it was centrifuged at 350× *g* for 3 min each time. Pig red blood cells are obtained when the supernatant is close to colorless and transparent after cleaning and centrifugation of porcine anticoagulant whole blood. PAM was spread onto a 96-well plate and 20 μL of 1% porcine red blood cells was added to each well. The sample was diluted to 10^−1^, 10^−2^, 10^−3^, 10^−4^, 10^−5^, 10^−6^, and 10^−7^ and inserted into a 96-well plate containing PAM and red blood cells. Eight repeat wells were set for each dilution. The adsorption of red blood cells was observed for 7 d. Fifty percent hemadsorption doses (HAD_50_) were calculated according to the Reed–Muench method [34].

### 2.13. Biosafety Statement and Facility

All ASF live virus experiments were carried out in the biosafety level 3 (P3) facility of Lanzhou Veterinary Research Institute of the Chinese Academy of Agricultural Sciences and approved by the Ministry of Agriculture and Rural Affairs and the China National Accreditation Service for Conformity Assessment

### 2.14. Statistical Analysis

The significance of the experimental results was analyzed using GraphPad Prism v.8 (San Diego, CA, USA). All data are presented as mean values ± standard errors from three independent experiments. *, *p* < 0.05 was considered statistically significant. **, *p* < 0.01 was considered highly statistically significant.

## 3. Results

### 3.1. Identification of MGF360-9L-Interacting Factors in PK-15 Cells by Liquid Chromatography–Mass Spectrometry

**Figure 1:**
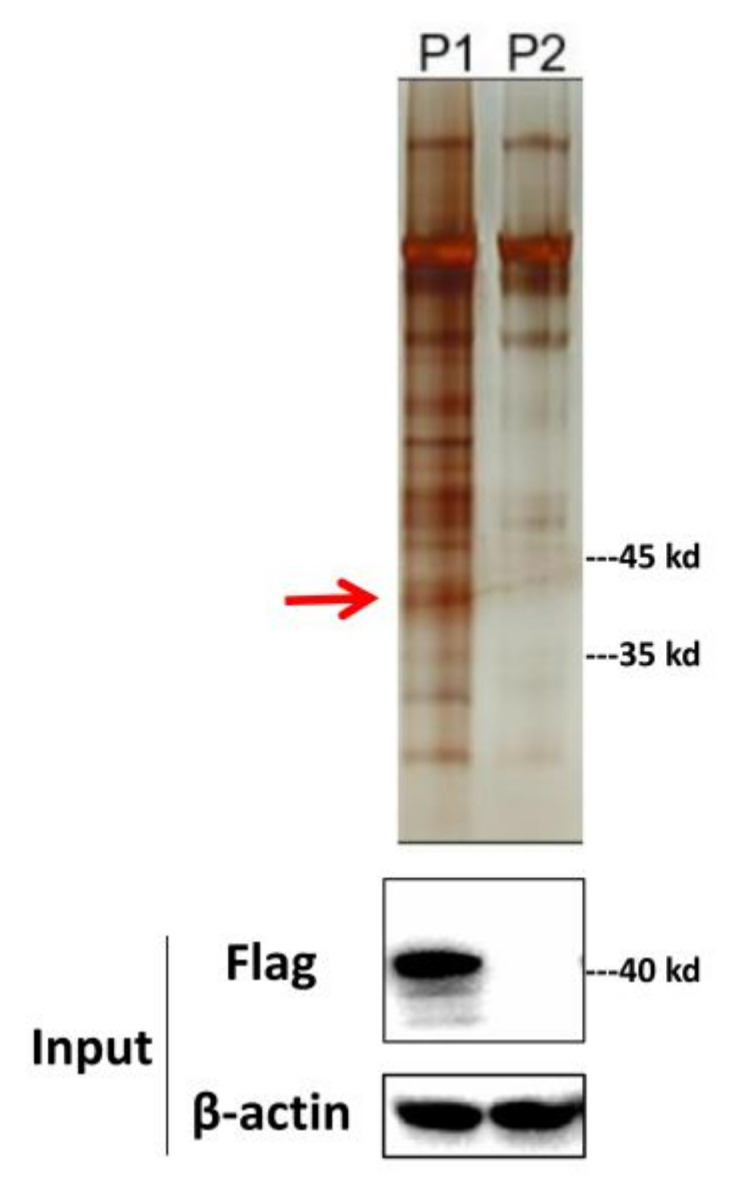
Western blotting and silver staining were used to detect the expression of exogenous MGF360-9L in PK-15 cells and the enrichment of MGF360-9L interacting proteins. Empty FLAG vector (8 μg) or FLAG-MGF360-9L (8 μg) was transfected into PK-15 cells. FLAG monoclonal antibody was used for pull down 24 h after transfection. MGF360-9L-interacting host proteins were eluted with protein A/G sepharose and analyzed on SDS-PAGE followed by silver staining. β-actin was used as a loading control. Lane P1, MGF360-9L protein complex, the MGF360-9L protein is represented by a red arrow; Lane P2, empty FLAG control. The data were tested three times independently.

To identify the potential host protein binding to MGF360-9L, the 3 × FLAG tag MGF360-9L plasmid and empty FLAG were transfected into PK-15 cells for co-immunoprecipitation and LC-MS analysis. The cell lysates were immunoprecipitated using anti-FLAG antibody and stained with silver to show MGF360-9L and its binding proteins. Immunoprecipitation samples of cells transfected with empty FLAG were used as negative controls to eliminate non-specific interactions. Compared with the negative control, the bands consistent with the molecular weight of Flag-MGF360-9L and the enrichment of MGF360-9L interacting partners were observed in the silver-stained samples (Figure 1).

LC-MS identified the interaction between MGF360-9L and PK-15 cell proteome. The final MGF360-9L binding protein data eliminated the nonspecific interaction through empty FLAG control. The interactions that remained were analyzed by significance analysis of interactome. Finally, 268 cellular proteins interacting with MGF360-9L were identified.

### 3.2. ASFV MGF360-9L-Host Interactome

**Figure 2:**
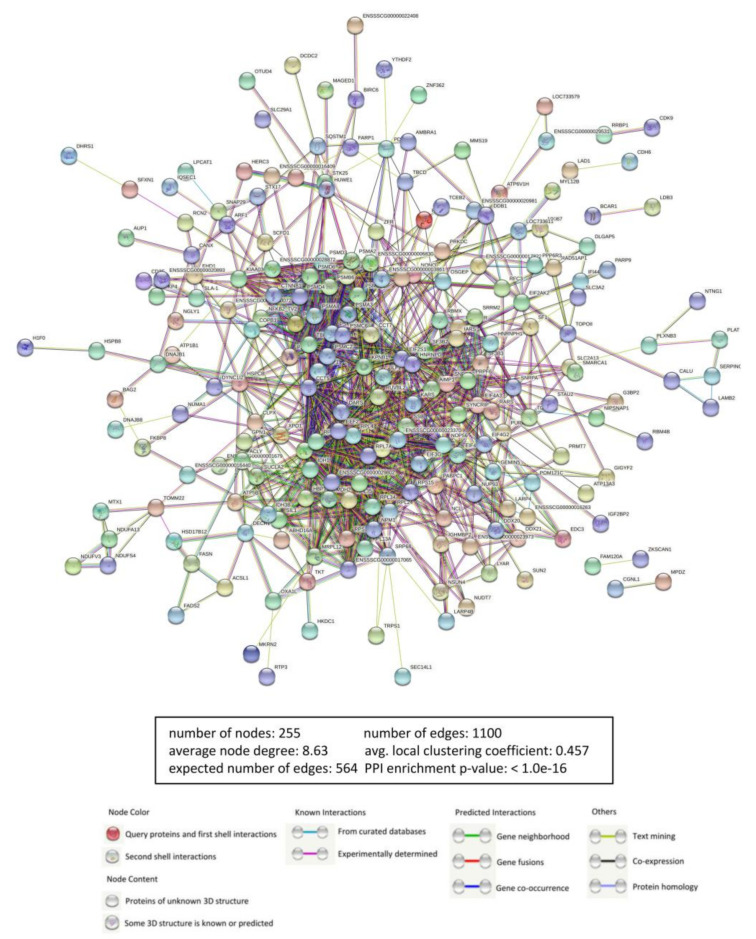
Construction and analysis of the protein–protein interaction (PPI) network using the STRING database. Each edge color indicates a different method of PPI prediction, as indicated below in the figure. The map of ASFV MGF360-9L-interacting proteins further interacting with the other proteins of our data was constructed and plotted using the network analyzer tool, Cytoscape v.3.7.1. The corresponding symbols indicating different protein classes are mentioned in the figure. Proteins are represented as the respective NCBI gene names.

Proteins are functional units in cells. The interaction network between MGF360-9L and cellular proteins was constructed using the STRING database to comprehensively analyze the protein interactions between MGF360-9L interacting host proteins (Figure 2). The observed number of edges for the network (1174) was significantly higher than expected for the given number of nodes (594). This means that many host proteins do not interact with MGF360-9L but bind to other host proteins to exert their biological functions in a functional protein complex form to participate in viral replication. Therefore, whether there is a direct interaction between MGF360-9L and these host proteins needs to be further verified. Details of host proteins interacting with MGF360-9L can be found in Supplementary Data S1.

### 3.3. GO Analysis

**Figure 3:**
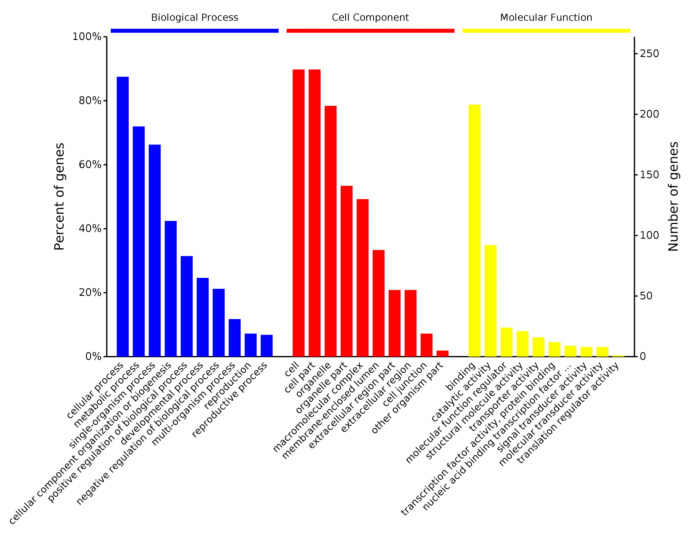
Gene ontology (GO) analysis based on the interaction between cell protein and MGF360-9L. Using Cytoscape v.3.7.1 and ClueGO software plug-in, the GO distributions of all proteins were divided into three types. The y were shown for significantly enriched terms based on biological process, molecular function (MF), and cellular component at *p* < 0.05.

GO analysis was performed on 268 MGF360-9L interacting proteins using the OmicsBean analysis tool to predict their biological functions (Figure 3). Biological processes, such as cellular processes, metabolic processes, single-organism processes, cellular component organization or biogenesis, positive regulation of biological processes, and developmental processes, were enriched. Furthermore, cells, cell parts, organelle, organelle parts, and macromolecular complexes were enriched under the cellular component and binding category. Catalytic activity and the molecular function regulator were enriched under the molecular function category. The GO annotation and analysis of all target proteins inferred that MGF360-9L might disrupt protein binding, metabolism, and innate immune response.

### 3.4. KEGG Pathway Enrichment Analysis

**Figure 4:**
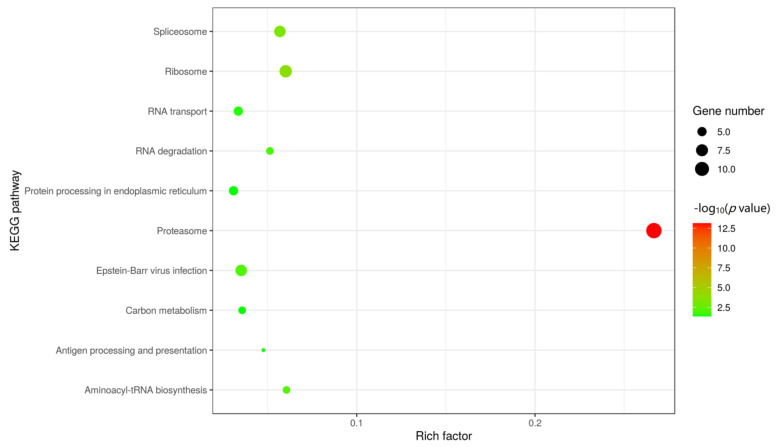
KEGG pathway enrichment analysis. The enriched pathways targeted by ASFV MGF360-9L-interacting proteins were analyzed using the KEGG functional annotation pathway database. The terms that were significantly enriched (*p* < 0.05) were shown.

To further understand and predict the pathway enrichment of proteins interacting between MGF360-9L and the host, we used KEGG to analyze the pathway enrichment and listed the top ten enrichment pathways (Figure 4). Interestingly, most of the target proteins are involved in proteasome, ribosome, spliceosome, Epstein-Barr virus infection, RNA degradation, and the carbon metabolism pathway. The proteasome pathway is closely related to virus infection, for example, an active ubiquitin proteasome system is necessary for efficient replication of infectious bronchitis virus in Vero cells [35,36]. TRIM21 inhibits porcine epidemic diarrhea virus proliferation by proteasomal degradation of the nucleocapsid protein [36]. Also, SARS-CoV-2 hijacks folate and one-carbon metabolism for viral replication [37]. The results suggest that these proteins play a potential role in ASFV infection.

### 3.5. Domain Enrichment Analysis of ASFV MGF360-9L-Interacting Partners

The abundance of protein domains can provide clues to the essential structure of interacting partners. To understand the binding preference of MGF360-9L, FunRich (http://funrich.org/index.html, accessed on 2 December 2020) was used to analyze protein domain enrichment of MGF360-9L-related interacting protein datasets. It can be speculated that cellular proteins containing AAA, proteasome_A_N, Iso_dh, and RRM domains tend to interact physically with MGF360-9L (Table 2).

### 3.6. Validating the Interactions between Cellular Proteins with MGF360-9L Protein, and MGF360-9L May Be Involved in Regulation of Host Metabolism by ASFV

**Figure 5:**
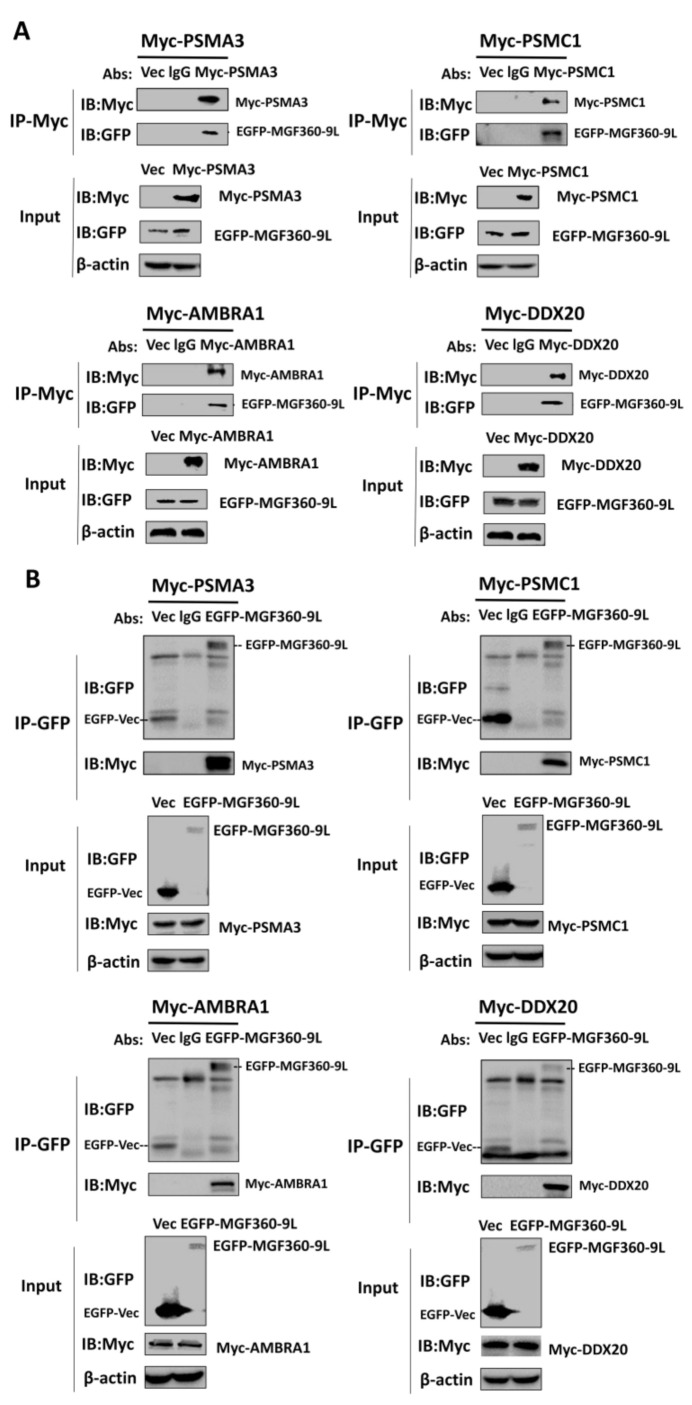
Verification of MGF360-9L-cell protein interaction. (**A**) HEK-293T cells were co-transfected with plasmids expressing Myc-PSMA3, Myc-PSMC1, Myc-AMBRA1, or Myc-DDX20, and plasmids expressing EGFP-MGF360-9L. EGFP-MGF360-9L was co-transfected with an empty vector as a negative control. Cell lysates were detected by immunoprecipitation with lgG and anti-Myc monoclonal antibodies, SDS-PAGE separation, and western blotting and detected with corresponding antibodies. β-actin was used as an internal loading control. (**B**) HEK-293T cells were co-transfected with plasmids expressing Myc-PSMA3, Myc-PSMC1, Myc-AMBRA1, or Myc-DDX20, and plasmids expressing EGFP-MGF360-9L. Plasmids expressing Myc-PSMA3, Myc-PSMC1, Myc-AMBRA1, or Myc-DDX20 and empty vector pCDNA3.1(+)-EGFP-C were used as a negative control. Cell lysates were detected by immunoprecipitation with lgG and anti-GFP monoclonal antibodies, SDS-PAGE separation, and western blotting and detected with corresponding antibodies. β-actin was used as an internal loading control.

To verify the protein interactions obtained after LC-MS and to determine whether MGF360-9L is involved in host metabolism regulation by ASFV, co-immunoprecipitation experiments were performed in vitro. Four cellular proteins were selected from MGF360-9L-interacting cellular proteins; these selected proteins were related to cell metabolism. To eliminate the effect of labeling on the authenticity of PPI, we selected different tagged plasmids EGFP-MGF360-9L (different from those used when preparing LC-MC samples) and empty vector PCDNA3.1(+)-Myc, Myc-PSMA3, Myc-PSMC1, Myc-AMBRA1, and Myc-DDX20 plasmid to co-transfect HEK-293T cells, then subjected them to immunoprecipitation with Myc monoclonal antibody (mAb) and GFP mAb, respectively. The results showed that MGF360-9L had specific interactions with PSMA3, PSMC1, AMBRA1, and DDX20, and no signal was observed in the empty vector control (Figure 5A,B).

**Figure 6:**
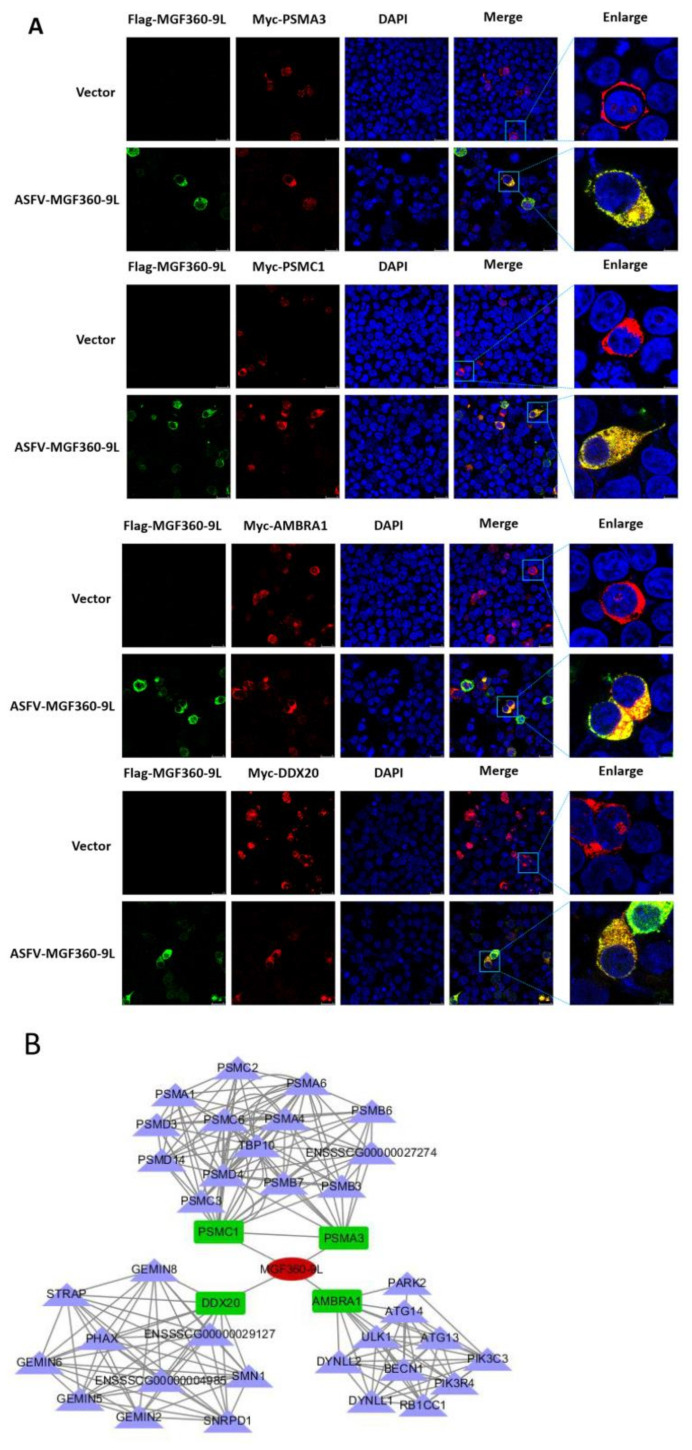
Colocalization of MGF360-9L with PSMA3, PSMC1, AMBRA1, and DDX20. (**A**) HEK- 293T cells were co-transfected with plasmids expressing Myc-PSMA3, Myc-PSMC1, Myc-AMBRA1, or Myc-DDX20 (1 μg), and plasmids expressing Flag-MGF360-9L (1 μg). After 24 h, the cells were treated by indirect immunofluorescence and observed by confocal microscope. Among them, the cells were incubated with anti-Flag rabbit mAb and anti-Myc mouse mAb for 8 h. (**B**) MGF360-9L-cellular protein interaction network. Cytoscape v.3.7.1 was used to construct the interaction map between ASFV MGF360-9L protein and interacting cellular protein. The data were tested three times independently.

To examine the colocalization of MGF360-9L protein with PSMA3, PSMC1, AMBRA1, and DDX20, HEK-293T cells were co-transfected with plasmids expressing Flag-MGF360-9L and Myc-PSMA3, Myc-PSMC1, Myc-AMBRA1, or Myc-DDX20. The results showed that MGF360-9L could colocalize with PSMA3, PSMC1, AMBRA1, and DDX20 by confocal microscopy (Figure 6A).

Overall, these data show that MGF360-9L interacts with PSMA3, PSMC1, and AMBRA1. The results also verified the data produced by proteomic analysis based on LC-MS. Another interaction network between MGF360-9L and cellular proteins confirmed by experiments was constructed (Figure 6B), which may help study the regulation of MGF360-9L on host metabolism and its potential role in the viral life cycle and the replication and pathogenesis of ASFV.

### 3.7. PSMA3 and PSMC1 Overexpression Significantly Promoted ASFV Replication, and MGF360-9L Maintained the Transcriptional Level of PSMA3 and PSMC1

**Figure 7:**
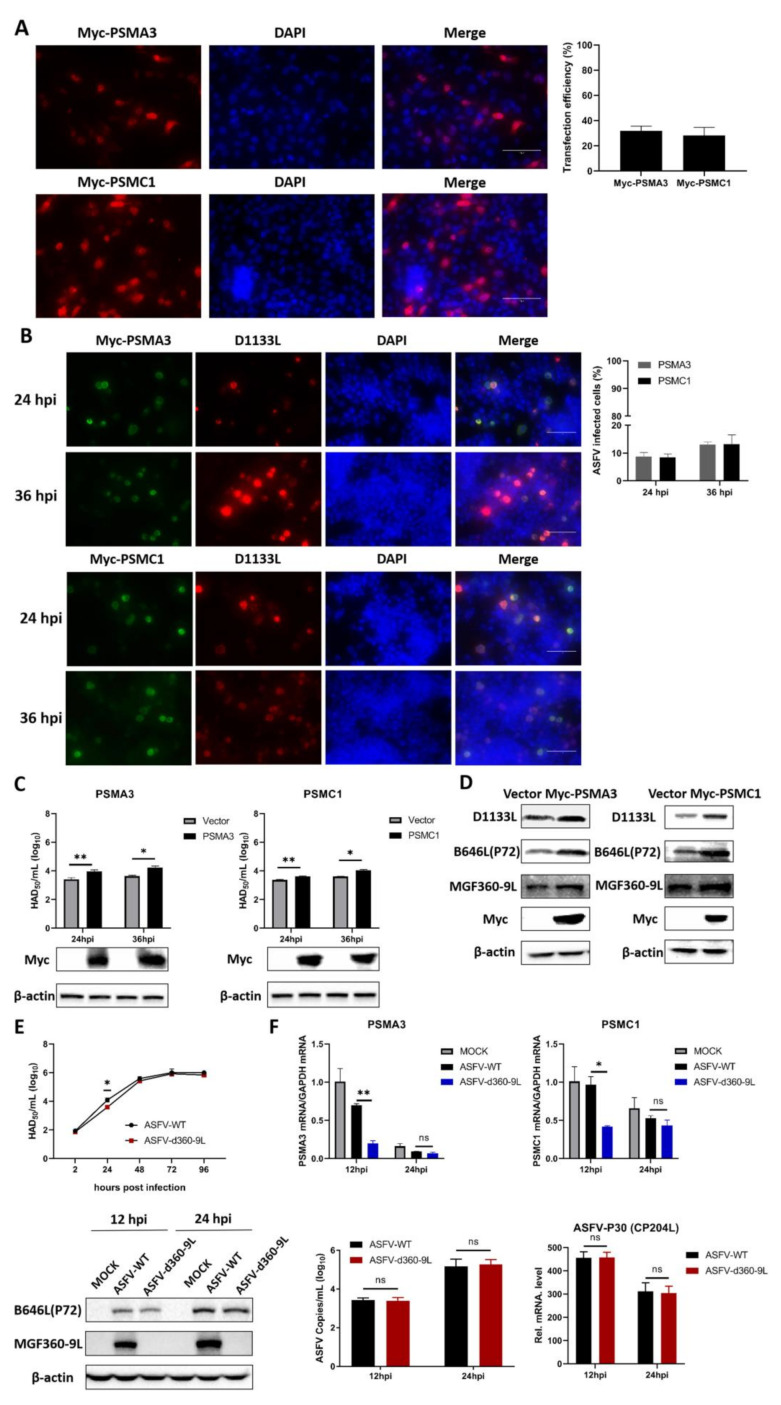
Overexpression host proteins PSMA3 and PSMC1 are beneficial to ASFV replication. (**A**) MA-104 cells were transfected with Myc-PSMA3 (4 μg/well) or Myc-PSMC1 (4 μg/well) plasmids. After 24 h, the transfection efficiency was observed by EVOS M5000 cell imaging system. (**B**) MA-104 cells were transfected with Myc-PSMA3 (4 μg/well) or Myc-PSMC1 (4 μg/well) plasmids. At 24 h after transfection, 0.1 MOI dose of ASFV was added to the cell. EVOS M5000 cell imaging system was used to observe whether ASFV infection occurred in the same cell expressing recombinant protein PSMA3 or PSMC1 and percentage of ASFV infected cells at 24 and 36 hpi. D1133L mAb is used to detect the expression of ASFV D1133L protein to refer to ASFV infection. (**C**) When the MA-104 cells were laid on 12-well plates, and the confluence degree reached 80%, empty vector (4 μg/well), Myc-PSMA3 (4 μg/well), or Myc-PSMC1 (4 μg/well) was transfected into the cells. At 24 h after transfection, 0.1 MOI dose of ASFV was added to the cell. The titers of ASFV were detected by HAD_50_ at 24 and 36 hpi. Expression of Myc-PSMA3 and Myc-PSMC1 was analyzed by western blotting. Statistical significance between groups was determined using *t*-test with GraphPad Prism v.8. *, *p* < 0.05 was considered statistically significant. **, *p* < 0.01 was considered highly statistically significant. ns: no significant difference. (**D**) When the MA-104 cells were laid on 12-well plates, and the confluence degree reached 80%, empty vector (4 μg/well), Myc-PSMA3 (4 ug/well), or Myc-PSMC1 (4 μg/well) was transfected into the cells. At 24 h after transfection, 0.1 MOI dose of ASFV was added to the cell. D1133L, B646L, and MGF360-9L protein expression was detected by western blotting at 24 hpi. β-actin was used as an internal loading control. (**E**) PAM was infected by ASFV-WT (MOI = 0.01) and ASFV-d360-9L (MOI = 0.01), and the titers of the virus were determined at 2, 24, 48, 72, and 96 hpi by HAD_50_ method. Statistical significance was determined using ANOVA and multiple comparisons. *, *p* < 0.05 was considered statistically significant. ns: no significant difference. (**F**) PAM cells were laid on a 6-well plate, and ASFV-WT (MOI = 0.1) or ASFV-d360-9L (MOI = 0.1) was added. The supernatant was discarded at 12 and 24 hpi, and the cells were collected. RNA was extracted and reverse transcribed. RT-qPCR was used to detect PSMA3 and PSMC1 gene transcription. GAPDH was used as an internal loading control. The same treated cell samples were collected to detect the protein expression of P72 and MGF360-9L. β-actin was used as an internal loading control. In addition, the same treated cell samples were collected to detect copy number of ASFV and mRNA level of early expressed genes P30 (CP204L) in ASFV. Statistical significance was determined using ANOVA and multiple comparisons. *, *p* < 0.05 was considered statistically significant. **, *p* < 0.01 was considered highly statistically significant. ns: no significant difference. Data of the mean ± S.D. of three independent experiments are shown.

PSMA3 and PSMC1 are the interaction factors of the ASFV MGF360-9L protein, and PSMA3 and PSMC1 are components involved in proteasome catabolism. PSMA3 or PSMC1 was co-transfected with MGF360-9L, and the results showed that PSMA3 and PSMC1 interacted with MGF360-9L (Figure 5 and Figure 6). To determine the effect of PSMA3 and PSMC1 on ASFV replication, Myc-PSMA3 or Myc-PSMC1 was transfected into the MA-104 cells. The transfection efficiency of Myc-PSMA3 and Myc-PSMC1 was 31.7% and 28.3%, respectively (Figure 7A). Twenty-four hours after transfection, 0.1 MOI dose of ASFV was added to the cells to observe whether ASFV infection occurred in the same cells expressing recombinant protein PSMA3 or PSMC1. The infection rate and titer of ASFV were further detected at 24 and 36 hpi. Results showed that ASFV infection is more likely to occur in MA-104 cells expressing recombinant protein PSMA3 or PSMC1(Figure 7B). The infection percentage of ASFV in MA-104 cells transfected with PSMA3 plasmid were 8.7% and 13.1%, at 24 and 36 hpi, respectively. The infection percentage of ASFV in MA-104 cells transfected with PSMC1 plasmid were 8.4% and 13.2%, at 24 and 36 hpi, respectively (Figure 7B). The viral titer of samples overexpressing PSMA3 or PSMC1 were significantly upregulated at 24 and 36 hpi (Figure 7C), indicating that PSMA3 and PSMC1 promotes ASFV replication. Western blotting also proves this point. At 24 hpi, the expression of ASFV protein D1133L, B646L, and MGF360-9L in overexpressed PSMA3 or PSMC1 samples was significantly higher than in the empty vector control (Figure 7D).

The growth curves of ASFV-WT and ASFV-d360-9L (deletion of MGF360-9L from ASFV-WT) were drawn by HAD_50_ methods. The results showed that the replication of ASFV-d360-9L was lower than that of ASFV-WT at 24 hpi. There was no significant difference in replication between the two viruses on PAM at 48 hpi (Figure 7E). To further explore the relationship between ASFV MGF360-9L, PSMA3, and PSMC1, the transcriptional levels of PSMA3 and PSMC1 in PAM infected with ASFV-WT (MOI = 0.1) or ASFV-d360-9L (MOI = 0.1) were detected. Results showed that at 12 hpi, the transcriptional levels of PSMA3 and PSMC1 in PAM infected with ASFV-d360-9L were significantly lower than in PAM infected with ASFV-WT (Figure 7F). MGF360-9L can maintain the transcription of PSMA3 and PSMC1 after ASFV infection.

## 4. Discussion

ASFV causes hemorrhagic diseases in pigs and wild boars with a high fatality rate [9]. As no effective commercial vaccine has been developed to control the spread of ASFV, the only way to control its transmission is to cull infected animals. Therefore, the spread of ASFV has brought huge losses and threatens the pig industry. ASFV is a large double-stranded DNA virus, which encodes many proteins [38]. Although much research has been conducted on the ASFV protein’s function, little is known about the molecular mechanism of ASFV replication and pathogenesis and its dependence on host factors. The interaction between ASFV and its host plays a vital role in its life cycle. Through complex interactions with host proteins, ASFV can evade the host’s defense system and escape innate immunity [39]. Also, many viruses, including foot-and-mouth disease virus (FMDV), strictly depend on the host–cell translation system to reproduce [40]. However, host cells also synthesize antiviral proteins to limit viral replication; for example, tripartite motif-containing 35 and eukaryotic translation elongation factor 1 delta can inhibit the replication of influenza A virus [41,42]. Viruses can participate in host cells’ biological processes through interaction with host proteins, therefore changing the microenvironment of host cells, inhibiting the synthesis of host proteins, and releasing cell resources to optimize their replication and transmission. For example, Zika and Dengue viral infections inhibit the stress response of host cells and ensure the synthesis of viral proteins [43]. Similarly, ASFV stimulates cap-dependent translation by activating the eIF4F complex to increase viral mRNA translation initiation [44]. Also, ASFV pE66L induces host translation shutdown through the PKR/eIF2α pathway [11].

Previous studies have shown that some genes in ASFV MGF360 can inhibit the host’s interferon production and play an essential role in the pathogenicity of the virus in pigs [19,24,25]. However, as a member of the MGF360 family, the role of MGF360-9L in viral infection has not been reported, and how MGF360-9L interacts with host proteins to regulate other host’s biological processes is also unknown. This study screened and identified 268 cellular proteins that may interact with ASFV MGF360-9L and drew a PPI network map. The number of sides of the observed network (1174) is significantly higher than expected (594), meaning there are more interactions than expected. Also, this enrichment indicated that many proteins aggregate into a complex or group to function. The biological function of the MGF360-9L-interacting protein was clarified by bioinformatics analysis. GO analysis showed that these proteins were expressed in cellular processes, metabolic processes, positive regulation of biological processes, and catalytic activity; also, molecular function regulators were enriched. KEGG pathway analysis showed that MGF360-9L-interacting protein was enriched in the proteasome, ribosome, spliceosome, and carbon metabolism pathway. The proteasome, ribosome, spliceosome, and carbon metabolism pathway enriched by MGF360-9L-cell protein interaction may play an essential role in ASFV infection. Previous studies have shown that the proteasome system is vital in ASFV replication [45]. Domain analysis of MGF360-9L-cell-interacting proteins showed that proteins with AAA, proteasome_A_N, Iso_dh, and RRM structure may tend to interact physically with MGF360-9L. Proteins with AAA structures are involved in various cellular processes, including membrane fusion, proteolysis, and DNA replication [46]. Many proteins with proteasome_A_N structure have protease function. Among the selected proteins interacting with MGF360-9L, PSMA1, PSMA2, PSMA3, and PSMA5 have a proteasome_A_N structure. Subsequently, we verified the interaction between MGF360-9L and PSMA3, PSMC1, AMBRA1, and DDX20, a selected cellular protein related to cell metabolism [47,48,49,50], in vitro by co-immunoprecipitation and IFA. Many viruses, such as Hepatitis B Virus and SARS-CoV-2, hijack the metabolic processes of host cells to escape cellular defense or to acquire substances needed for replication [51,52]. GO, KEGG, and domain analyses of interacting proteins showed that MGF360-9L likely regulates host metabolic response during ASFV infection.

Overexpressed proteins, PSMA3 and PSMC1, involved in proteasome catabolism, promote ASFV replication. This result supports the previous research that the proteasome system plays a crucial role in ASFV replication [45]. However, it is unknown whether PSMA3 and PSMC1 promote ASFV replication based on their role in the proteasome pathway. Also, we cannot use PAM (the natural ASFV host cell) to prepare LC-MC samples or study the effect of overexpressed host protein on ASFV replication because the PAM’s transfection efficiency is low. In the experiment on the effect of overexpressed host proteins on ASFV replication, MA-104 cells, recently found to be infected by ASFV [53], were used. The LC-MC samples were prepared by PK-15 cells to obtain the data related to MGF360-9L interacting with host cells. We found that the replication level of ASFV with MGF360-9L deletion (ASFV-d360-9L) was lower than that of the parent strain at 24 hpi in vitro, and there was no difference in replication between ASFV-d360-9L and the parent strain after 48 hpi. The transcriptional level of PSMA3 and PSMC1 in PAM infected with ASFV-WT and ASFV-d360-9L was significantly different at 12 hpi, and the difference disappeared at 24 hpi. This indicates that the MGF360-9L protein maintains PSMA3 and PSMC1 transcription during ASFV infection. The cause of the absence of the transcriptional difference between PSMA3 and PSMC1 in PAM infected with ASFV-WT and ASFV-d360-9L at 24 hpi is uncertain. We speculate that ASFV encodes approximately 170 proteins; furthermore, the interaction between viral proteins is complex. The interaction between ASFV and the host is also complex, and there may be more than one viral protein that targets PSMA3 and PSMC1. Previous studies have shown that many viral proteins can degrade essential cellular defenses and immune proteins through the proteasome pathway, conducive to viral replication. Pseudorabies virus UL24 degrades IRF7 through the proteasome pathway and antagonizes IFN-β mediated activation by cGAS-STING [54]. Foot-and-mouth disease virus VP1 interacts with TPL2 and degrades TPL2 through the proteasome pathway to inhibit TPL2-mediated antiviral activity [55]. As a large double-stranded DNA virus encoding many proteins, ASFV can degrade essential cellular defenses and immune proteins through the proteasome pathway. Based on existing data, it is speculated that ASFV MGF360-9L may degrade some critical cellular defense and immune proteins through the proteasome pathway to promote viral replication.

## 5. Conclusions

In this study, the interaction between MGF360-9L and cellular proteins was systematically screened for the first time in PK-15 cells transfected with MGF360-9L. We identified 268 proteins that might interact with MGF360-9L. The PPI network was constructed, and the potential function of identified host proteins was predicted by GO and KEGG enrichment analyses. In the co-immunoprecipitation experiment, all four proteins related to the metabolic process could bind to MGF360-9L. Overexpression of PSMA3 or PSMC1 protein is beneficial to ASFV replication. Understanding cellular proteins and interference pathways targeted by ASFVMGF360-9L may contribute to a comprehensive understanding of viral–host interactions and provide new insights into new targets for identifying host effects and viral factors. Our data also suggest that MGF360-9L regulates host–cell biological processes through the proteasome pathway, but further studies are needed. Also, future studies in pigs are necessary to evaluate the role of MGF360-9L in ASFV infection.

## Figures and Tables

**Table 1:** Primers and oligonucleotides used in this study.

Primers	Sequences (5′-3′)
Porcine PSMA3-F	GTGGATAAAGGGGACGCCAT
Porcine PSMA3-R	AGCTGCTCCAGTCTGTTTCC
Porcine PSMC1-F	GCCAAAGCAAGATGGGTCAAA
Porcine PSMC1-R	GGCACTGAGTGTGAGGTGTC
ASFV P30 (CP204L)-F	CTCCGATGAGGGCTCTTGCT
ASFV P30 (CP204L)-R	AGACGGAATCCTCAGCATCTTC
Porcine GAPDH-F	ACATGGCCTCCAAGGAGTAAGA
Porcine GAPDH-R	GATCGAGTTGGGGCTGTGACT

**Table 2:** Enriched protein domains of ASFV MGF360-9L-PK-15 cell-interacting proteins.

Protein Domain	*p* Value
AAA	6.23311 ×10^−10^
Proteasome_A_N	3.42772 × 10^−6^
Iso_dh	3.35825 × 10^−5^
RRM	0.000561107
PP2Ac	0.000877893
small_GTPase	0.001859956
S4	0.002218629
KH	0.003060462
KOW	0.00602828
coiled coil region	0.008893437
ARM	0.009385491
PHB	0.011492043
RAN	0.015154577
OMPdecase	0.015154577
CRM1_C	0.015154577

## Data Availability

All datasets generated for this study are included in the article/Supplementary Material. The mass spectrometry proteomics data have been deposited to the ProteomeXchange Consortium via the PRIDE partner repository with the dataset identifier PXD027260.

## Supplementary Materials

The following are available online at https://www.mdpi.com/article/10.3390/v13091804/s1, Supplementary data S1: Host protein interacting with MGF360-9L.
